# Case Report: Atypical cat-scratch disease presenting as referred shoulder pain and deep lymphadenopathy in a pediatric patient

**DOI:** 10.3389/fped.2026.1933692

**Published:** 2026-09-28

**Authors:** Qi-hang Jiang, Qun Zhu, Zhu-jie Xu, Qing-bo Chen, Yuan-hua Cen

**Affiliations:** Department of Pediatrics, The People’s Hospital of Fenghua Ningbo, Ningbo, China

**Keywords:** atypical manifestations, *Bartonella henselae*, cat scratch disease, children, deep lymphadenopathy, shoulder pain

## Abstract

**Background:**

Cat-scratch disease (CSD) is a zoonotic infection attributable to *Bartonella henselae*. Typical clinical manifestations include regional lymphadenopathy accompanied by primary cutaneous lesions that develop following exposure to cats. Although most patients present with typical regional lymphadenopathy, a subset of patients may develop atypical manifestations, which can complicate diagnosis. Owing to the nonspecific clinical features, CSD is prone to misdiagnosis or diagnostic omission. This report describes a pediatric case of atypical cat-scratch disease characterized by initial symptoms of fever and shoulder arthralgia, absence of superficial lymphadenopathy, and involvement of deep lymph nodes. This report aims to broaden clinicians’ recognition of the diverse and atypical clinical manifestations of this disease.

**Case summary:**

An 11-year-old female patient presented to the hospital with persistent fever accompanied by left shoulder joint pain and restricted movement of the left upper extremity. Physical examination upon admission revealed no superficial lymphadenopathy or characteristic cutaneous lesions. Following empirical broad-spectrum antibiotic therapy, the patient continued to experience recurrent febrile episodes, with persistently elevated inflammatory markers. Subsequent magnetic resonance imaging (MRI) of the shoulder joint and contrast-enhanced computed tomography (CT) of the chest demonstrated multiple enlarged lymph nodes in the left axillary, supraclavicular, and deep cervical regions. Further history-taking disclosed recent exposure to cats. Histopathological examination of an axillary lymph node biopsy (hematoxylin–eosin staining) revealed suppurative granulomatous lymphadenitis. These H&E histopathological findings are merely supportive rather than confirmatory evidence for CSD, as we failed to perform *Bartonella henselae*-specific serology, PCR, Warthin–Starry silver staining or immunohistochemistry to identify the pathogen directly. The diagnosis was considered highly suggestive of atypical CSD based on integrated clinical, radiological, epidemiological, and histopathological findings. Upon diagnosis, the patient was administered doxycycline in combination with rifampicin for antimicrobial treatment. The patient's body temperature normalized, shoulder pain progressively resolved, and inflammatory markers returned to reference ranges. During a 3-month follow-up period, no disease recurrence was noted. Shoulder joint mobility was restored to normal, and no significant adverse drug reactions were observed.

**Conclusion:**

In pediatric patients presenting with fever of unknown origin accompanied by musculoskeletal symptoms, suboptimal response to conventional anti-infective therapy, and absence of superficial lymphadenopathy, the possibility of atypical cat scratch disease should be considered. A detailed assessment of contact history with domestic animals, along with prompt deep lymph node imaging evaluation and histopathological biopsy, facilitates early and appropriate clinical diagnosis. Implementation of targeted antimicrobial therapy may improve clinical outcomes in affected children.

## 1 Introduction

Cat-scratch disease (CSD) is a zoonotic infection caused by *Bartonella henselae* (1), predominantly observed in pediatric and adolescent populations. Its characteristic clinical manifestations include cutaneous inoculation-site lesions and regional superficial lymphadenopathy, with most cases following a self-limiting and benign clinical course (2). However, approximately 5%–25% of patients exhibit atypical presentations affecting the skeletal system, nervous system, ocular tissues, or deep-seated lymphadenopathy (3). Owing to the lack of pathognomonic clinical features, atypical CSD is frequently misdiagnosed as purulent infections, autoimmune diseases, or neoplasms (4). Recognition of CSD remains particularly challenging in children presenting with fever and musculoskeletal manifestations in the absence of superficial lymphadenopathy. Such atypical presentations frequently lead clinicians to exclude CSD from the initial differential diagnosis, resulting in considerable delays in diagnosis and the initiation of appropriate therapy. This report describes a case of atypical cat-scratch disease in a pediatric patient who presented with shoulder pain and fever in the absence of superficial lymphadenopathy. The diagnosis was revised based on magnetic resonance imaging findings revealing deep lymph node involvement, which was further supported by histopathological findings from lymph node biopsy. By summarizing the clinical manifestations, diagnostic workup and treatment regimen of this case, we aim to raise clinicians’ awareness of atypical cat-scratch disease, underscore the supportive clinical value of deep lymph node imaging, and provide references for early suspicion and standardized management of comparable cases.

**Table 1:** Timeline of diagnosis and treatment for this pediatric patient.

Course	Major Events
D0	Fever developed (maximum temperature 39.0 °C), accompanied by left shoulder pain and restricted range of motion.
D0-D3	The patient received oral cefaclor and intravenous cefmetazole in the outpatient setting, but symptoms did not improve.
D4	The patient was admitted to the pediatric department because of persistent fever.
D4	Admission laboratory tests: CRP 76.8 mg/L, ESR 28 mm/h, PCT 0.12 ng/mL
D4–D9	Despite the administration of ceftriaxone as empirical antimicrobial therapy, Recurrent fever persisted throughout the treatment period, with a maximum tympanic temperature of 38.8 °C. Fever could be temporarily controlled by antipyretics, yet temperature rebounded every 6 to 8 h.
D8	Shoulder MRI revealed multiple enlarged lymph nodes in the left axillary and supraclavicular regions.
D9	Contrast-enhanced chest CT confirmed multiple enlarged deep lymph nodes involving the left cervical, supraclavicular, and axillary regions.
D10	Axillary lymph node biopsy demonstrated suppurative granulomatous lymphadenitis, and the hematoxylin–eosin (H&E) morphology was suggestive of cat scratch disease.
D10	Antimicrobial therapy was adjusted to oral doxycycline 100 mg twice daily combined with rifampicin 300 mg twice daily. That afternoon, the patient's body temperature began to decline, with a reduced fever peak of 37.9 °C.
D11	Body temperature returned to normal, and left shoulder pain gradually improved.
D18	The patient was discharged with clinical improvement and continued oral therapy to complete a total treatment course of 14 days.
Three months after discharge	No recurrence of fever was observed. Shoulder function returned to normal, and no obvious adverse drug reactions were reported.

D0 indicates the first day of symptom onset, and D represents the number of days elapsed since symptom onset.

## 2 Case description

The patient, an 11-year-old female weighing 49 kg, was admitted to the Department of Pediatrics at Fenghua District People's Hospital with a chief complaint of “fever accompanied by left shoulder pain for 4 days.”

Four days prior to admission, the patient developed fever of unknown etiology, with a maximum recorded body temperature of 39.0 °C, accompanied by chills and rigors during febrile episodes. Concurrently, she experienced left shoulder pain and restricted mobility of the left upper extremity, with symptoms exacerbated by movement. No associated symptoms such as cough, dyspnea, vomiting, diarrhea, or rash were reported, and there was no documented history of trauma. Outpatient laboratory investigations revealed an elevated C-reactive protein (CRP) level of 30.8 mg/L, while complete blood count showed no significant abnormalities. Based on these findings, bacterial infection was suspected, and sequential antimicrobial therapy with oral cefaclor followed by intravenous cefmetazole was initiated. However, due to persistent fever and shoulder pain, the patient was hospitalized for further diagnostic evaluation and management.

The patient had an unremarkable past medical history, with normal growth and development, no previous illnesses or surgical interventions, and no relevant family medical history.

On admission, physical examination revealed a body temperature of 37.8 °C, respiratory rate of 22 breaths per minute, pulse of 90 beats per minute, and blood pressure of 92/60 mmHg. The patient was alert and oriented. Mild pharyngeal congestion was observed, along with grade I enlargement of bilateral tonsils. No palpable enlargement of superficial lymph nodes was detected in the neck, axillae, or other regions, and no skin rashes or excoriations were present. Significant localized tenderness was noted over the left shoulder joint without overlying erythema, warmth, or swelling, and active elevation of the left upper limb was limited; the right shoulder was normal, rendering the finding unilateral and asymmetric. The remainder of the physical examination was unremarkable. Upon hospital admission, laboratory investigations revealed: C-reactive protein (CRP) level of 76.8 mg/L (reference range: 0–10 mg/L), erythrocyte sedimentation rate (ESR) of 28 mm/h (reference range: 0–20 mm/h), and procalcitonin (PCT) level of 0.12 ng/mL (reference range: <0.05 ng/mL). Blood cultures, respiratory pathogen testing, tuberculin test (PPD), T-SPOT.TB and rheumatoid-related indicators were all negative. Electrocardiogram (ECG) and hepatic and splenic ultrasonography revealed no abnormalities. Given the persistent fever and shoulder pain, bacteremia, septic arthritis, and other infectious diseases were preliminarily suspected.

Following admission, the child continued to receive intravenous ceftriaxone as antimicrobial therapy. However, fever recurred post-treatment, and left shoulder pain demonstrated no significant alleviation. Re-evaluation on the fourth day of admission indicated a CRP level of 40.2 mg/L (reference range: 0–10 mg/L), an ESR of 44 mm/h (reference range: 0–20 mm/h), and no significant abnormalities in routine blood tests. Due to the poor efficacy of conventional antimicrobial therapy, further magnetic resonance imaging (MRI) of the left shoulder was conducted. The MRI revealed no abnormalities in the shoulder joint or surrounding bones but indicated multiple enlarged lymph nodes in the left axillary and clavicular regions. Subsequently, contrast-enhanced chest computed tomography (CT) (Figure 1) confirmed multiple enlarged lymph nodes in the left neck, supraclavicular region, and deep axillary area.

**Figure 1:**
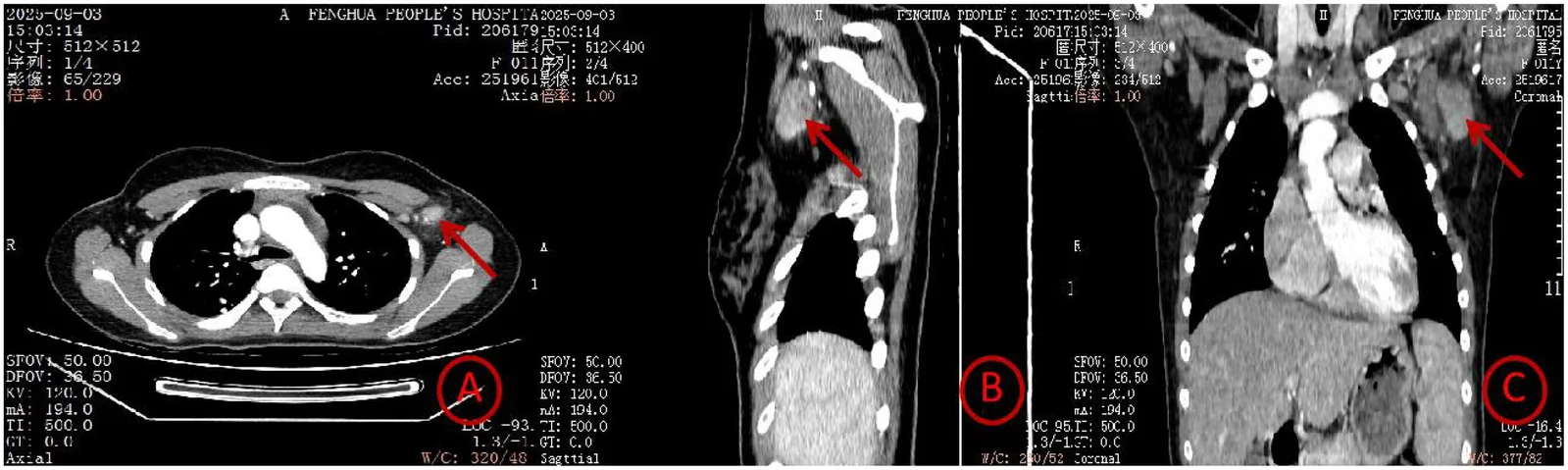
Multiplanar contrast-enhanced chest CT demonstrating deep lymphadenopathy in atypical cat-scratch disease. Contrast-enhanced chest CT images showing multiple enlarged lymph nodes in the left supraclavicular and axillary regions (red arrows). **(A)** Axial view; **(B)** sagittal reconstruction; **(C)** coronal reconstruction.

In light of the imaging findings, a thorough re-assessment of the medical history disclosed that the patient had direct contact with stray cats two weeks before symptom onset. Accordingly, an ultrasound-guided core needle biopsy of the axillary lymph nodes was performed. Histopathological examination demonstrated the presence of granulomatous suppurative lymphadenitis; these pathological findings provided supportive evidence consistent with cat-scratch disease (Figure 2).

**Figure 2:**
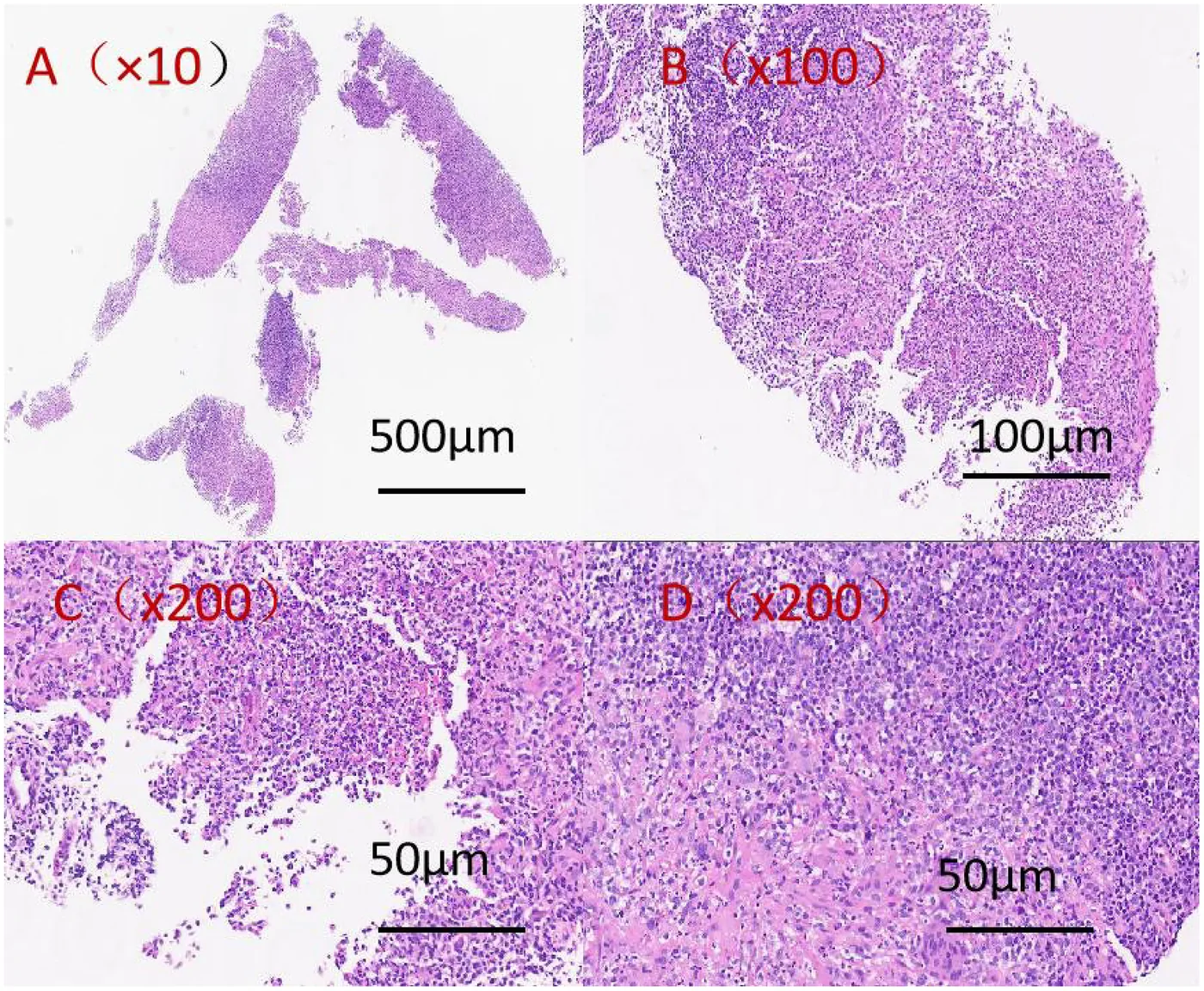
Histopathological findings of the biopsied lymph node. **(A)** Low-power view showing partial effacement of lymph node architecture (H&E staining, scale ba*r* = 500*μ*m). **(B)** Intermediate-power view demonstrating stellate microabscess formation surrounded by inflammatory cells (scale ba*r* = 100μm). **(C)** High-power view showing prominent neutrophilic infiltration within microabscesses (scale ba*r* = 50μm). **(D)** High-power view showing multinucleated giant cells and proliferating monocytoid B cells adjacent to suppurative lesions (scale ba*r* = 50μm).

Regrettably, confirmatory tests specific for *Bartonella henselae* were not conducted in this case, including serum antibody testing, PCR-based amplification of *Bartonella* genes, Warthin-Starry silver staining, and immunohistochemical staining of biopsy specimens. The paraffin-embedded lymph node tissue block from the patient was discarded upon expiration of the standard pathological archive retention period, thereby precluding retrospective molecular pathogen detection. Stellate suppurative granulomatous lymphadenitis observed via hematoxylin-eosin staining provides only suggestive pathological evidence and is insufficient to independently confirm *Bartonella henselae* infection.

The initial clinical presentation, characterized by persistent fever, elevated inflammatory markers, and shoulder pain, was consistent with a suppurative infection. However, the absence of clinical response to *β*-lactam therapy and the lack of magnetic resonance imaging (MRI) findings supportive of joint infection prompted a reassessment of the preliminary diagnostic hypothesis. The detection of deep lymphadenopathy emerged as a critical diagnostic turning point. Synthesis of the patient's animal contact history, deep lymph node involvement, supportive histopathological features, and exclusion of alternative differential diagnoses supported a clinical diagnosis of atypical cat-scratch disease. Following diagnostic clarification, the therapeutic regimen was revised to combination antimicrobial therapy with doxycycline hydrochloride 100 mg (approximately 2.04 mg/kg per dose) orally twice daily and rifampicin 300 mg (approximately 6.12 mg/kg per dose) orally twice daily. After initiation of the doxycycline plus rifampicin regimen at the above dosages, the patient's body temperature gradually normalized, with complete defervescence achieved by the 11th day of illness. Concurrently, left shoulder pain and associated limitations in mobility demonstrated progressive improvement. By the 18th day of illness, clinical recovery was sufficient to permit discharge, after completion of a 14-day course of anti-infective therapy. During a three-month post-discharge follow-up period, no recurrence of fever was observed, full range of motion was restored in the left shoulder joint, and C-reactive protein (CRP) levels returned to the normal range. No significant drug-related adverse events were reported throughout the treatment or follow-up intervals.

Given the atypical presentation, several infectious, rheumatologic, and malignant conditions were considered during the diagnostic process (Table 2).

**Table 2:** Differential diagnoses considered in the present case and reasons for exclusion.

Differential diagnosis	Findings supporting the diagnosis	Findings arguing against the diagnosis/Reasons for exclusion
Septic arthritis	Fever, shoulder pain, elevated inflammatory markers, limitation of shoulder movement	MRI showed no joint effusion, synovial thickening, or destructive changes of the shoulder joint; symptoms did not improve after prolonged *β*-lactam antibiotic therapy
Osteomyelitis	Fever accompanied by musculoskeletal pain and elevated CRP/ESR	MRI revealed no evidence of bone marrow edema, cortical destruction, or osteomyelitis-related changes
Bacteremia	Persistent fever with increased inflammatory markers	Blood cultures remained negative, PCT was only mildly elevated (0.12 ng/mL), and there was no response to broad-spectrum β-lactam antibiotics
Juvenile idiopathic arthritis (JIA)	Shoulder pain with restricted movement	Acute onset with high fever, absence of chronic arthritis manifestations, negative rheumatologic investigations, and subsequent identification of infectious etiology
Lymphoma	Deep cervical, supraclavicular, and axillary lymphadenopathy	Histopathological examination demonstrated suppurative granulomatous lymphadenitis rather than malignant infiltration; rapid clinical improvement was observed following targeted antimicrobial treatment
Tuberculosis lymphadenitis	Persistent fever, deep lymphadenopathy, granulomatous inflammation on lymph node biopsy	Negative PPD and T-SPOT.TB tests; no evidence of pulmonary tuberculosis on chest CT; histopathology demonstrated suppurative granulomatous inflammation with stellate microabscess formation rather than typical caseating granulomas
Non-tuberculous mycobacterial lymphadenitis	Pediatric age group, lymph node enlargement, granulomatous inflammation	No chronic unilateral cervical lymphadenopathy, no characteristic skin discoloration or sinus formation, acute febrile course and rapid response to doxycycline/rifampicin favored CSD
Brucellosis	Fever of unknown origin, elevated inflammatory markers, possible osteoarticular manifestations	No history of exposure to livestock or unpasteurized dairy products; no typical systemic manifestations; clinical response and lymph node pathology were inconsistent with brucellosis
Syphilis	Lymphadenopathy and systemic inflammatory manifestations may occur in secondary syphilis	No characteristic skin lesions or epidemiological risk factors; histopathological findings were not compatible with syphilitic lymphadenitis
Final integrated diagnosis	Atypical cat-scratch disease (clinically diagnosed based on integrated epidemiological, clinical, radiological, and histopathological evidence)	-
Cat-scratch disease	Cat exposure history; persistent fever; deep lymphadenopathy on MRI and contrast-enhanced CT; suppurative granulomatous lymphadenitis with stellate microabscesses on H&E histopathology (supportive evidence only); rapid clinical response to doxycycline + rifampicin	Qualifying features: no superficial lymphadenopathy and no typical inoculation-site lesion, consistent with atypical CSD presentation; definitive microbiological confirmation (PCR, serology, Warthin–Starry staining) was not performed

## 3 Discussion

Cat scratch disease (CSD) is a zoonotic infection attributable to *Bartonella henselae* (1), with adolescents representing the predominant high-risk population (2, 5). The primary modes of transmission are cat scratches and bites, which allow infected feline saliva to come into contact with broken skin. The disease typically manifests as primary skin lesions accompanied by regional superficial lymphadenopathy, often following a self-limited clinical course (2)^.^ However, atypical manifestations of CSD, which may occur without the characteristic skin lesions or superficial lymphadenopathy, can pose significant diagnostic challenges. Herein, we report the case of an 11-year-old girl who presented with fever and shoulder pain as the initial symptoms in the absence of superficial lymphadenopathy, representing an atypical presentation of CSD with deep lymph node involvement. This case highlights the importance of considering CSD in children with unexplained systemic symptoms and atypical clinical presentations.

Previous studies have shown that pediatric cat-scratch disease can manifest with musculoskeletal complications, such as osteomyelitis and arthritis (6, 7); Nevertheless, most cases typically present with regional superficial lymphadenopathy (8). In the present case, the child presented with shoulder pain in the absence of superficial lymphadenopathy. The diagnosis was subsequently established incidentally via magnetic resonance imaging (MRI) and contrast-enhanced computed tomography (CT) of the chest, which revealed abnormalities in the deep lymph nodes. Such presentations are infrequent and indicate that the clinical spectrum of atypical cat-scratch disease may be broader than previously recognized.

In the present case, the child exhibited pronounced left shoulder pain and restricted mobility of the left upper extremity, yet MRI revealed no abnormalities in the shoulder joint or surrounding osseous structures. Contrast-enhanced CT identified multiple enlarged lymph nodes in the left axillary, supraclavicular, and deep cervical regions. This dissociation between the intensity of subjective pain and the absence of intra-articular pathology strongly implicates a neuropathic or referred pain mechanism mediated by deep lymph node involvement. Anatomically, the axillary lymph nodes lie in close proximity to the cords of the brachial plexus; enlargement of these nodes may exert direct mechanical compression on the brachial plexus, producing pain along the C5–T1 dermatomal distribution that encompasses the shoulder region. The supraclavicular and deep cervical lymph nodes are situated adjacent to the cervical nerve roots (C3–C5) and the phrenic nerve—structures with well-established roles in mediating referred pain to the shoulder. A systematic review by Fernández-López et al. demonstrated that afferent fibers of the phrenic nerve (originating from C3–C5) converge onto the same spinal dorsal horn neurons that receive nociceptive input from the shoulder dermatomes, thereby providing a neuroanatomical substrate for referred shoulder pain originating from deep thoracic and cervical structures (9). The convergence-projection theory and the dichotomizing afferent fiber hypothesis, recently reviewed by Jin et al., offer complementary mechanistic explanations: sustained nociceptive input from inflamed deep lymph nodes may induce central sensitization of spinal dorsal horn neurons, such that afferent signals from the shoulder region are perceptually amplified and localized as shoulder pain (10). Furthermore, the granulomatous suppurative inflammation characteristic of cat-scratch disease releases pro-inflammatory mediators, including tumor necrosis factor-α, interleukin-1β, and interleukin-6, which can directly sensitize perineural nociceptors and lower the activation threshold of adjacent sensory nerve fibers. The temporal concordance between the initiation of targeted antimicrobial therapy and the subsequent resolution of shoulder pain in the present case substantiates the causal relationship between deep lymphadenopathy and shoulder symptomatology. Collectively, the available evidence suggests that deep lymph node enlargement in the axillary–supraclavicular–cervical chain can precipitate shoulder pain through a combination of mechanical compression of adjacent neural structures, inflammatory sensitization of nociceptive afferents, and central mechanisms of referred pain. Clinicians evaluating children with unexplained shoulder pain should maintain a high index of suspicion for deep lymph node pathology, particularly when articular imaging is unremarkable and inflammatory markers are persistently elevated.

The initial differential diagnosis in this patient included bacterial suppurative infections, granulomatous lymphadenitis caused by infectious pathogens, autoimmune diseases, and malignancies (Table 2). Particular consideration was given to tuberculosis and other infectious causes of suppurative granulomatous lymphadenitis because the histopathological findings were not specific for cat-scratch disease. Given the lack of efficacy of empirical *β*-lactam antibiotic therapy and the absence of MRI-detected joint or bone infection, the diagnostic approach was further refined. The MRI finding of deep lymph node involvement constituted a pivotal juncture in the diagnostic process, with subsequent histopathological findings further supporting the diagnosis of atypical cat scratch disease.

Diagnostic criteria for cat scratch disease (CSD) includ e(11): (1) A history of repeated exposure to cats or dogs, accompanied by scratches, bites, or visible inoculation lesions; (2) Positive findings on specific antigen skin testing or specific antibody detection assays; (3) Aspiration of pus from enlarged lymph nodes with subsequent culture and laboratory testing to rule out alternative causative pathogens; (4) Lymph node biopsy revealing pathognomonic histopathological changes. Traditionally, a definitive diagnosis of CSD is established if three out of these four criteria are met.

In this clinical case, criteria 1, 3 and 4 were satisfied: the patient had a clear history of close contact with cats, deep lymphadenopathy with other etiologies excluded via laboratory testing, and lymph node histopathological features suggestive of cat-scratch disease. Notably, histopathological changes constitute supportive rather than confirmatory evidence for CSD, as suppurative granulomatous lymphadenitis is not pathognomonic and may be observed in other granulomatous infectious diseases. Criterion 2 (specific antigen skin test or serological antibody testing) was not conducted at our medical center. Collectively, the comprehensive clinical, radiological and histopathological findings are highly suggestive of atypical cat-scratch disease. Nevertheless, given the lack of direct microbiological verification, the clinical diagnosis was reached through integrated assessment of epidemiological history, clinical manifestations, imaging findings, and supportive histopathological features rather than definitive pathogen identification. Previous studies have demonstrated that even advanced imaging modalities, including PET-CT, may not reliably distinguish benign from malignant lymph node lesions in certain cases (12). Therefore, when microbiological confirmation is unavailable, histopathological examination remains an important component of the diagnostic evaluation. The diagnosis of cat-scratch disease (CSD) requires a comprehensive interpretation of clinical manifestations, epidemiological history, imaging findings, and pathological characteristics. Typical histopathological features include stellate microabscess formation and suppurative granulomatous inflammation within lymph nodes (13), which may assist in differentiating CSD from other conditions such as lymph node tuberculosis, metastatic malignancy, and necrotizing lymphadenitis. However, suppurative granulomatous lymphadenitis is not specific to CSD and may also occur in other granulomatous infectious diseases; therefore, pathological findings should always be interpreted in the context of clinical presentation and exclusion of alternative diagnoses.

In the present case, the diagnosis of atypical CSD was supported by three major findings: a history of close feline exposure, deep lymphadenopathy identified by imaging, and characteristic lymph node histopathological changes. The diagnosis was further strengthened by the consistency among clinical features, radiological findings, pathological results, and therapeutic response. This case highlights the importance of considering atypical CSD in pediatric patients with unexplained fever and musculoskeletal symptoms, particularly when symptoms persist despite empirical antimicrobial therapy. Clinicians should actively investigate potential animal exposure and consider targeted imaging and biopsy of deep lymph nodes when conventional diagnostic approaches fail to identify the underlying cause.

The pediatric patient presented with persistent high fever and shoulder joint pain, in the absence of superficial lymphadenopathy, culminating in a diagnosis of atypical cat-scratch disease (CSD). Despite administration of multiple *β*-lactam antibiotics, no significant clinical or laboratory improvement was observed. Consequently, the therapeutic regimen was adjusted to incorporate a combination of doxycycline and rifampicin (14). Following this intervention, the patient achieved afebrile status, and the shoulder pain progressively resolved. The administration of tetracycline-class antibiotics in pediatric populations has been historically contraindicated owing to the associated risk of dental discoloration (15). Nevertheless, emerging evidence suggests that short-term doxycycline therapy does not appear to be associated with a significantly increased risk of discoloration in permanent dentition (15). The patient in this case was an 11-year-old pediatric individual who presented with persistent high-grade fever, extensive lymphadenopathy, and refractoriness to conventional therapeutic interventions. The treatment regimen was selected based on a comprehensive evaluation of the disease severity relative to the pharmacological safety profile. The patient exhibited favorable tolerance to the therapy, suggesting that doxycycline, in combination with rifampicin and guided by individualized assessment, may constitute a feasible therapeutic approach for atypical pediatric cat scratch disease (CSD). However, additional investigations are warranted to substantiate its efficacy.

Currently, there are no internationally standardized treatment guidelines for cat scratch disease (CSD), and high-quality evidence-based medical data remain limited (14). In typical CSD cases involving immunocompetent patients with localized lymphadenitis, the majority of infections are self-limiting and are managed mainly with symptomatic and supportive measures. A randomized, double-blind, placebo-controlled trial conducted by Bass et al. (16) demonstrated that azithromycin promotes short-term lymph node reduction without improving final clinical outcomes. Therefore, the principal benefit of antimicrobial therapy in typical cat scratch disease lies in alleviating symptoms rather than altering the natural course of the disease. In contrast, atypical or disseminated Bartonella infections may involve critical organs, including the eyes, central nervous system, bones, and heart. The CDC and NIH recommend aggressive antimicrobial therapy for these patients, commonly selecting drugs such as doxycycline and rifampicin, which exhibit intracellular activity (17, 18). However, high-quality evidence supporting the optimal treatment regimen and duration remains insufficient.

This study is subject to several limitations. First, and most importantly, no *Bartonella henselae*-specific microbiological confirmation — including serology, polymerase chain reaction (PCR), Warthin–Starry silver staining, or immunohistochemistry — was performed at our institution. The diagnosis therefore rests on integrated clinical, imaging, and histopathological evidence rather than on direct pathogen identification. As the paraffin-embedded tissue block from the index biopsy is no longer available, retrospective molecular analysis cannot now be performed. We recommend that future similar cases include PCR-based detection (targeting the 16S rRNA or gltA gene) or Warthin–Starry silver staining of biopsy material whenever available to achieve definitive microbiological confirmation. Second, as a single-case report, the generalizability of the findings necessitates further validation through additional cases and prospective studies. Finally, the follow-up period was restricted to three months, indicating that the long-term prognosis and safety of the doxycycline and rifampicin combination therapy require continued observation. Nevertheless, this case provides a detailed description of the diagnostic process for atypical cat-scratch disease in children, offering valuable clinical insights for the early identification, standardized diagnosis, and management of similar pediatric cases.

## 4 Conclusion

In conclusion, this atypical presentation of cat scratch disease (CSD) in a pediatric patient, characterized by shoulder pain as the initial symptom and an absence of superficial lymphadenopathy, suggests that the clinical spectrum of CSD in children may be more diverse than previously recognized. For pediatric patients presenting with unexplained fever concomitant with joint symptoms who are unresponsive to conventional *β*-lactam antibiotic therapy, CSD should be included in the differential diagnosis, even in the absence of typical cutaneous lesions and superficial lymphadenopathy. Radiographic abnormalities in deep lymph nodes can provide critical diagnostic insights; when integrated with a comprehensive history of animal exposure and histopathological examination, this approach facilitates the prevention of misdiagnosis, promotes early and accurate therapeutic intervention, and enhances prognostic outcomes.

## Patient perspective

A formal patient or parent perspective was not obtained for this case report. Although the patient's parents/legal guardians provided written informed consent for publication, a structured assessment of their experience, expectations, or perception of the diagnostic and treatment process was not conducted.

## Data Availability

The original contributions presented in the study are included in the article/Supplementary Material, further inquiries can be directed to the corresponding author.
